# Highly pathogenic avian influenza subtype H5Nx clade 2.3.4.4 outbreaks in Dutch poultry farms, 2014–2018: Clinical signs and mortality

**DOI:** 10.1111/tbed.13597

**Published:** 2020-05-17

**Authors:** Janneke Schreuder, Thijs T. M. Manders, Armin R. W. Elbers, Arco N. van der Spek, Ruth J. Bouwstra, J. Arjan Stegeman, Francisca C. Velkers

**Affiliations:** ^1^ Department of Farm Animal Health Faculty of Veterinary Medicine Utrecht University Utrecht the Netherlands; ^2^ Department of Bacteriology and Epidemiology Wageningen Bioveterinary Research Lelystad the Netherlands; ^3^ Netherlands Food and Consumer Product Safety Authority (NVWA) Utrecht the Netherlands; ^4^ GD Animal Health Deventer the Netherlands

**Keywords:** H5N6 subtype, H5N8 subtype, influenza A virus, mortality, poultry, signs and symptoms

## Abstract

In recent years, different subtypes of highly pathogenic avian influenza (HPAI) viruses caused outbreaks in several poultry types worldwide. Early detection of HPAI virus infection is crucial to reduce virus spread. Previously, the use of a mortality ratio threshold to expedite notification of suspicion in layer farms was proposed. The purpose of this study was to describe the clinical signs reported in the early stages of HPAI H5N8 and H5N6 outbreaks on chicken and Pekin duck farms between 2014 and 2018 in the Netherlands and compare them with the onset of an increased mortality ratio (MR). Data on daily mortality and clinical signs from nine egg‐producing chicken farms and seven Pekin duck farms infected with HPAI H5N8 (2014 and 2016) and H5N6 (2017–2018) in the Netherlands were analysed. In 12 out of 15 outbreaks for which a MR was available, MR increase preceded or coincided with the first observation of clinical signs by the farmer. In one chicken and two Pekin duck outbreaks, clinical signs were observed prior to MR increase. On all farms, veterinarians observed clinical signs of general disease. Nervous or locomotor signs were reported in all Pekin duck outbreaks, but only in two chicken outbreaks. Other clinical signs were observed less frequently in both chickens and Pekin ducks. Compared to veterinarians, farmers observed and reported clinical signs, especially respiratory and gastrointestinal signs, less frequently. This case series suggests that a MR with a set threshold could be an objective parameter to detect HPAI infection on chicken and Pekin duck farms at an early stage. Observation of clinical signs may provide additional indication for farmers and veterinarians for notifying a clinical suspicion of HPAI infection. Further assessment and validation of a MR threshold in Pekin ducks are important as it could serve as an important tool in HPAI surveillance programs.

## 1 INTRODUCTION

In recent years, different subtypes of highly pathogenic avian influenza A (HPAI) viruses have caused outbreaks in different poultry types worldwide (Lee, Bertran, Kwon, & Swayne, 2017; Napp, Majó, Sánchez‐Gónzalez, & Vergara‐Alert, 2018).

Clearly, early detection of HPAI virus infection on poultry farms is essential to reduce risks for virus spread and minimize the socio‐economic impact of the disease (Backer, van Roermund, Fischer, van Asseldonk, & Bergevoet, 2015; Elbers, Fabri, et al., 2004), which is also increasingly reflected in legislation and contingency plans worldwide. European Union legislation on the control of HPAI (EU, 2005a, 2005b) stipulates that early detection systems, aimed at a rapid reporting of any sign of avian influenza in poultry and other captive birds by owners or keepers to the competent veterinary authority, need to be in place. For both LPAI and HPAI outbreaks, sudden changes in mortality have shown to be an indicator of infection (Elbers, Holtslag, Bouma, & Koch, 2007; Gonzales & Elbers, 2018; Malladi, Weaver, Clouse, Bjork, & Trampel, 2011), as well as clinical signs (Elbers, Kamps, & Koch, 2004; Elbers, Koch, & Bouma, 2005; Velkers et al., 2006).

These indicators have been used to formulate criteria in European Union legislation for reporting suspicion of a notifiable disease such as avian influenza in poultry, with even more detailed criteria implemented in national regulations in the Netherlands (Box 1). However, the current reporting thresholds may not be sensitive enough for timely detection of HPAI virus infections (Gonzales & Elbers, 2018). Published reports on analyses of mortality data from previous outbreaks, that is HPAI H7N7 in 2003 (Bos et al., 2007; Stegeman et al., 2004) and HPAI H5N8 in 2014 and 2016 (Velkers, Elbers, Bouwstra, & Stegeman, 2015) have shown that (a) it takes several days after the start of increased mortality due to HPAI virus infections to reach the official reporting threshold of 0.5% mortality for two consecutive days; and (b) many flocks have already been depopulated well before reaching these thresholds. To improve sensitivity of detection of LPAI and HPAI virus infections and at the same time maintain a high level of specificity, Gonzales and Elbers (2018) developed new reporting thresholds based on increased mortality and drops in egg production for layer farms, and evaluated the performance of those indicators with HPAI H7N7 outbreak data from 110 infected layer flocks in the Netherlands in 2003. The mortality ratio (MR), with a reporting threshold of 2.9 times higher mortality than the average weekly mortality of the previous week for that particular flock, had a 95.3% sensitivity to signal HPAI virus infection in laying hens and would have resulted in 2 days earlier detection compared with the current Dutch national thresholds for HPAI and in 7 days earlier detection for LPAI virus infection (Gonzales & Elbers, 2018).

Box 1 **European legislation and Dutch regulations on reporting criteria for avian influenza detection**

***European Commission Decision 2005/734/EC*** (EU, 2005a):

Article 2 stipulates that Member States shall introduce early detection systems, aimed at a rapid reporting of any sign of avian influenza in poultry and other captive birds by the owners or keepers to the competent veterinary authority.

Annex II: criteria to be considered when applying the measure set out in Article 2: drop in feed and water intake higher than 20%; drop in egg production higher than 5% for more than two days; mortality rate higher than 3% in a week; and any clinical sign or post‐mortem lesion suggesting avian influenza.

***Dutch Ministerial Regulation TRCJZ/2005/1411 concerning the prevention, control and monitoring of infectious animal diseases, zoonoses and transmissible spongiform encephalopathies (TSEs), Article 84*** (Dutch State Journal, 2005):

Poultry keepers have to report increased mortality in layers, reproduction birds or broilers (older than 10 days) to the authorities in case of 0.5% mortality or more per flock per day for two consecutive days; in turkeys in case of 1% mortality or more per day for two consecutive days; and in AI susceptible birds in case of 3% or more mortality per week.

Poultry keepers of AI susceptible birds need to consult their veterinarian in case of a clinical problem; reduction in feed intake or water intake of 5% or more per day for two consecutive days; in layers and breeders a reduction in egg production of 5% or more per day for two consecutive days.

**Approved veterinary programme of the Netherlands under EU Regulation 652/2014** (EC, 2019; Elbers, Gorgievski‐Duijvesteijn, Zarafshani, & Koch, 2010):

Additionally, to ensure timely detection and minimize spread of infections with low pathogenic avian influenza (LPAI) viruses, that can mutate to HPAI viruses, an intensive monitoring program that includes all commercial poultry holdings in The Netherlands is in place. Because LPAI virus infections can be asymptomatic or might generate only mild symptoms, veterinarians in the Netherlands can submit cloacal or pharyngeal swabs to exclude LPAI virus infection as a possible cause for clinical problems.

For early detection of HPAI virus infections, the suggested MR ratio threshold of 2.9 may also be applicable to other poultry types. Ssematimba et al. (2019) recently explored efficacy of mortality‐based triggers for HPAI virus detection in game birds, but for commercial ducks and turkeys, which are also commonly affected during HPAI outbreaks, mortality thresholds have not yet been evaluated. Furthermore, as clinical signs have proven to be indicators of HPAI virus infections, taking both MR and clinical signs into account may potentially further enhance early detection in different poultry types.

Therefore, the aim of this study was to describe the clinical signs reported in the early stages of HPAI H5N8 and H5N6 outbreaks on chicken and Pekin duck farms between 2014 and 2018 in the Netherlands and compare them with the onset of an increased MR. For this purpose, we collected data on mortality, production characteristics and clinical signs from 16 HPAI (H5N8 and H5N6) outbreaks on poultry farms between 2014 and 2018 in the Netherlands. We calculated the MR and daily mortality for each outbreak and provide an extensive inventory of the species‐specific clinical signs and how these developed over time in the days before official notification, as observed by poultry farmers and veterinarians.

## 2 METHODS

### 2.1 Data collection

**FIGURE 1:**
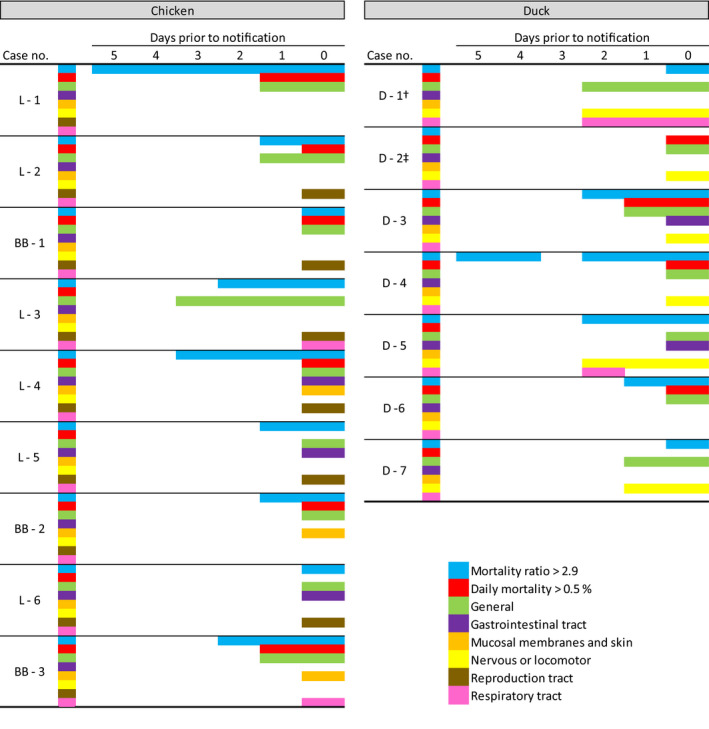
Clinical signs observed by the farmers, categorized by organ system, for the highly pathogenic avian influenza virus‐infected chicken (left) and duck farms (right) and exceedance of daily mortality (>0.5%) and mortality ratio (MR) thresholds in the 5 day period prior to notification. ^†^Day of notification for D‐1 was the day a positive result was found in the early warning swabs sent in by the veterinary practitioner. ^‡^Not enough mortality data were available to calculate the mortality ratio

A case series study was performed on a total of 16 poultry farms that were diagnosed with HPAI infection caused by viruses of subtypes H5N8 or H5N6 in the Netherlands between 2014 and 2018, which included six farms with laying hens, three farms with broiler breeders and seven farms with Pekin ducks (Table 1). The only other HPAI H5N8 outbreak in this period (World Organisation for Animal Health (OIE), 2017) not included in the analysis, was a wild water bird trading farm, that also housed domestic poultry in 2016. The day of notification (Table 1; Figure 1) refers to the day when the farmer or the veterinary practitioner reported a suspicion of avian influenza to the Netherlands Food and Consumer Product Safety Authority (NVWA). Only for outbreak D‐1, samples were submitted to the national reference laboratory by the veterinary practitioner in the Dutch national diagnostic framework of excluding LPAI (as described in Box 1; EC, 2019). In this outbreak, we considered the day of the positive result of these swabs as day of notification. In all outbreaks, a team consisting of a (state) veterinarian of the NVWA, a poultry veterinarian from GD Animal Health, and in most outbreaks the veterinary practitioner, visited the farms within 9 hr after notification for clinical inspection and official sample collection (referred to as veterinary inspection visit [VIV]). Inquiries on the history of the clinical situation observed by the farmer and clinical signs observed by the veterinarians during this inspection were recorded in a standardized form (see Section 2.3). Twenty cloacal and pharyngeal swabs were collected per flock. Swabs were tested at the national reference laboratory by PCR for antigen detection (Beerens et al., 2018). According to the protocol of HPAI virus‐positive farms, NVWA performed an epidemiological investigation to trace dangerous contacts prior to culling. This included a standardized interview with the farmer and collection of charts with at least daily records of mortality and production data, for example feed and water intake, and egg production. All birds on the HPAI virus‐positive farms were culled within 1–2 days after the day of notification (Table 1).

**TABLE 1:** Highly pathogenic avian influenza (HPAI) virus‐infected commercial chicken and duck farms in the Netherlands between 2014 and 2018 included in the study: notification and culling dates, flock data and HPAI virus subtype

Outbreak no.^a^	Date of notification	Date of culling	Poultry type	Flock size	Affected houses/total houses	Flock age at notification	HPAI virus type^b^
L‐1	14‐Nov‐14	16‐Nov‐14	Laying hens	124,000	1/6	55 weeks	H5N8
L‐2	19‐Nov‐14	21‐Nov‐14	Laying hens	41,400	1/3	67 weeks	H5N8
BB‐1	20‐Nov‐14	21‐Nov‐14	Broiler breeders	11,000	1/2	61 weeks	H5N8
D‐1^e^	21‐Nov‐14^c^	22‐Nov‐14	Pekin ducks	14,500	1/2	18 days	H5N8
L‐3^f^	29‐Nov‐14	30‐Nov‐14	Laying hens	28,000	1/1	22 weeks	H5N8
D‐2	25‐Nov‐16	26‐Nov‐16	Pekin ducks	10,000	1/1	40 days	H5N8
D‐3	30‐Nov‐16	1‐Dec‐16	Pekin ducks	8,500	1/1	24 days	H5N8
D‐4^g^	1‐Dec‐16	2‐Dec‐16	Pekin ducks	15,400	2/2	15 and 43 days^d^	H5N8
L‐4	12‐Dec‐16	14‐Dec‐16	Laying hens	63,000	1/3	38 weeks	H5N8
D‐5^e^	16‐Dec‐16	17‐Dec‐16	Pekin ducks	14,000	1/2	23 days	H5N8
L‐5	17‐Dec‐16	19‐Dec‐16	Laying hens	28,500	1/2	25 weeks	H5N8
BB‐2	19‐Dec‐16	20‐Dec‐16	Broiler breeders	48,000	1/4	30 weeks	H5N8
L‐6^f^	24‐Dec‐16	25‐Dec‐16	Laying hens	28,000	1/1	52 weeks	H5N8
D‐6^g^	7‐Dec‐17	8‐Dec‐17	Pekin ducks	16,000	1/2	29 days	H5N6
BB‐3	24‐Feb‐18	26‐Feb‐18	Broiler breeders	39,100	1/3	31 weeks	H5N6
D‐7^e^	12‐Mar‐18	13‐Mar‐18	Pekin ducks	29,700	1/2	32 days	H5N6

^a^ Outbreaks on Laying hen (L), Broiler Breeder (BB) and Duck (D) farms.

^b^ Diagnosis of HPAI, tested positive on real‐time PCR on the matrix gene, H5‐PCR and sequencing of the haemagglutinin and neuraminidase (Beerens et al., 2018).

^c^ Samples were submitted to the national reference laboratory by the veterinary practitioner in the framework of the Dutch early‐warning system, we considered the day of the positive result of these samples as day of notification.

^d^ Two flocks infected with HPAI virus present on the farm, one flock age 15 days the other age 43 days.

^e^ D‐1, D‐5 and D‐7 are outbreaks of HPAI on the same duck farm.

^f^ L‐3 and L‐6 are outbreaks of HPAI on the same laying hen farm.

^g^ D‐4 and D‐6 are outbreaks of HPAI on the same duck farm.

Additionally, an in‐depth epidemiological investigation was performed by specialized poultry veterinarians of the Faculty of Veterinary Medicine of Utrecht University. This investigation was performed for all farms between 9 days to 3 months after culling and was aimed to facilitate retrospective identification of the most likely moment and route of HPAI virus introduction and/or spread (referred to as Detailed Epidemiological Investigation [DEI]). For all farms, all available data collected by NVWA and laboratory results were evaluated, additional in‐depth interviews with farmers and farm employees, veterinarians from NVWA, GD Animal Health and the farms' veterinary practitioner were conducted retrospectively, and detailed production records were gathered. The farmers and veterinarians were inquired in detail about the course of infection and observed clinical and post‐mortem signs in the 2 weeks prior to notification up to and including the day of the VIV. These data were used for further data analyses as described below.

### 2.2 Mortality and production parameters

Mortality ratio (MR) and egg production ratio (EPR) were calculated as described by Gonzales and Elbers (2018) for each of the flocks, using available flock records of at least 5 days to approximately 1 month before notification. The threshold of 2.9 for MR, as applied for laying hens by Gonzales and Elbers (2018), was used and the first day the MR exceeded the threshold was considered as an increase in MR and used for further analyses. We were not able to calculate the MR for one Pekin duck farm (D‐2) due to incomplete mortality data in the weeks prior to the outbreak. The current applied daily mortality (DM) threshold of 0.5% per flock (see Box 1) was also used for comparisons. In layer farms, an EPR of below 0.94 was considered as presence of reproduction tract signs. The use of this threshold alone, and in combination with the MR, was validated as a way to detect LPAI and HPAI outbreaks at an early stage by Gonzales and Elbers (2018). Data on daily growth were not recorded in any of the affected farms. In farms where records of water and feed intake were available, a decrease in feed or water intake of 5% compared with the previous day was classified under general clinical signs as described below.

### 2.3 Clinical signs

The standardized form used to record clinical signs observed during the VIV included a yes or no checklist with questions on feed and water intake, sudden death, ruffled feathers, diarrhoea, egg quality, oedema and cyanosis, nervous signs, abnormal conjunctivae, lacrimation, respiratory distress and decreased activity. Furthermore, the veterinarians recorded findings on mortality, production and feed and water intake based on the flock records if available at time of VIV. At the DEI, poultry veterinarians of GD Animal Health and the farms' veterinary practitioners were questioned in more detail on the clinical signs on day of notification. In two outbreaks, the veterinary practitioner had visited the farm prior to notification, that is for outbreak D‐1 2 days and for BB‐3 1 day prior to notification. The observed clinical signs did not differ from the clinical signs observed at day of the VIV (data not shown). The farmers were queried at the DEI on the clinical signs observed in the period between 14 days prior to and the day of culling, but only the data until day of notification were used for the analyses. Also, the flock records were checked for notes on clinical signs.

A list of clinical signs, categorized in different categories, was used to compile all the data from the veterinarians from VIV and DEI, and only from de DEI for the farmers separately. The observed clinical signs were categorized in six categories, that is as clinical signs attributed to nervous and locomotor system; mucosal membranes and skin; respiratory tract; gastrointestinal tract; and reproduction tract (Tables S1–S4) or as general clinical signs. The latter category included signs of general illness, which could not be related to a specific organ system or were associated with signs of systemic disease, for example depression, reduced feed or water intake, ruffled feathers or hunched posture, cold or warm extremities and sudden death (Tables S1–S4). Signs of the nervous and locomotor system were categorized together as these were difficult to distinguish based on the information from the farmers. Mucosal membranes and skin signs included discolorations or oedema, most likely because of the endothelial damage caused by the virus, for example cyanosis, oedema and haemorrhages, including those in the conjunctivae. Excessive lacrimation and conjunctivitis without haemorrhages were categorized under (upper) respiratory signs. Decreased egg production (EPR < 0.94) and abnormal eggs were classified as signs of the reproduction tract. These data were used to report the frequency of detection of clinical signs for each of the six categories in Pekin duck and chicken farms (layers and broiler breeders) and for veterinarians and farmers separately.

## 3 RESULTS

### 3.1 Outbreaks

Five, eight and three farms were infected in the autumn‐winter period of 2014, 2016 and 2017–2018, respectively. No outbreaks occurred in 2015. In 2014 and 2016 six farms with laying hens, two broiler breeder farms and five Pekin duck farms were infected with HPAI virus H5N8. In the winter of 2017–2018, two Pekin duck farms and a broiler breeder farm tested positive for HPAI virus H5N6. Some farms were affected repeatedly. This was the case for Pekin duck farms D‐1, D‐5 and D‐7, for D‐4 and D‐6 and for laying hen flocks L3 and L6. On 13 farms, more than one poultry house was present, but only in one duck farm (D‐4) two houses tested HPAI virus positive. The age of infected Pekin ducks varied between 15 and 43 days and chicken flocks were between 22 and 67 weeks of age at notification.

### 3.2 Clinical signs

A detailed list of the observed clinical signs by farmers and veterinarians in the chicken and duck flocks based on VIV and DEI is provided in Tables S1–S4.

#### 3.2.1 Chicken farms

Figure 1 summarizes the clinical signs that were observed by farmers in their flocks in the 5 day period prior to notification to the authorities, and occurrences where the current official DM threshold for reporting (>0.5%) or the MR threshold (>2.9) were exceeded. In both parameters, the first day the parameter exceeded its threshold was used in the further analyses.

For the chicken farms, the first signs observed by the farmers were those of general disease in outbreak L‐3 at 3 days prior to notification. On the day of notification, the farmers of the chicken farms (*n* = 9 outbreaks) observed general clinical signs in all nine outbreaks, clinical signs of the reproduction tract in six outbreaks, clinical signs of mucosal membranes and skin in three outbreaks, clinical signs of the gastrointestinal tract in three outbreaks, and clinical signs of the respiratory tract in two outbreaks. MR exceeded the threshold in all nine outbreaks, but only in six outbreaks the DM exceeded 0.5% per day on day of notification.

**FIGURE 2:**
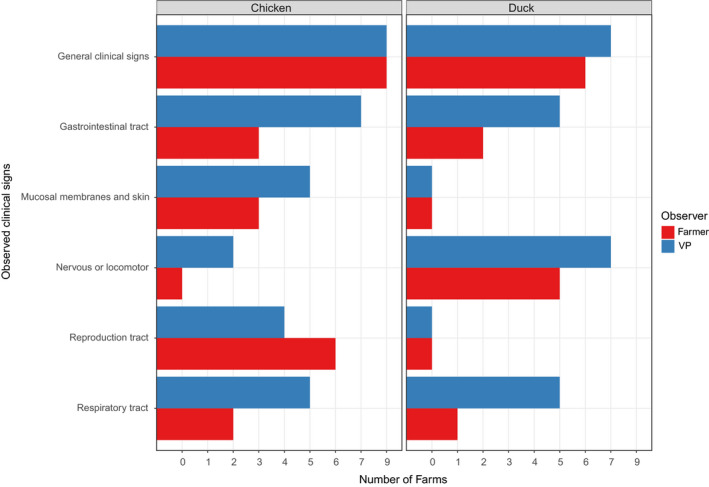
Overview of the frequency of detection of clinical signs, as categorized by organ system, observed on day of notification by farmers (in red) and veterinarians (in blue) on the day of veterinary inspection on highly pathogenic avian influenza virus‐infected chicken (*n* = 9, left) and duck farms (*n* = 7, right)

The frequency of observed clinical signs on the chicken farms (*n* = 9 outbreaks), as reported by the farmers (at day of notification) or veterinarians (during the VIV) for the six different categories is summarized in the left part of Figure 2. Similar to the farmers, the veterinarians reported general clinical signs in all nine chicken outbreaks. The frequency of the clinical signs reported by the veterinarians was higher for signs of the gastrointestinal tract (seven outbreaks), mucosal membranes and skin (five outbreaks), and respiratory tract (five outbreaks), but lower for reproduction tract (four outbreaks) compared with the farmers. None of the farmers reported nervous or locomotor signs, whereas veterinarians reported this in two outbreaks.

#### 3.2.2 Pekin duck farms

In the Pekin duck farms (*n* = 7 outbreaks), the first clinical signs were observed 2 days prior to the notification in two outbreaks (D‐1 and D‐5), which included general clinical signs and signs of the nervous or locomotor system and respiratory tract (Figure 1). For outbreak D‐5, temporary sneezing was only observed at day two before notification. A day prior to notification farmers observed clinical signs of the nervous or locomotor system in three, and of the respiratory tract in one of the outbreaks. On the day of notification, general clinical signs were observed in all seven outbreaks, signs of the nervous or locomotor system in six, gastrointestinal signs in two and respiratory tract signs in one of the outbreaks. The DM exceeded the 0.5% threshold in only four outbreaks whereas the MR exceeded the threshold of 2.9 in six outbreaks at day of notification. For D–2, the DM was only available from 1 day prior to notification and therefore the MR could not be calculated.

The frequency of observed clinical signs on the seven duck farms, as reported by the farmers (at day of notification) or veterinarians (during the VIV) for the six different categories are summarized in the right part of Figure 2. Overall, the frequency of clinical signs reported by the veterinarians was higher compared with the frequency of the clinical signs reported by the farmer. Similar to the farmers, the most prominent signs reported by the veterinarians were general clinical signs. Clinical signs of the nervous or locomotor system were observed in all seven Pekin duck outbreaks, and clinical signs of the respiratory tract and the gastrointestinal tract were observed in five outbreaks. In contrast with the veterinarians, farmers only reported respiratory signs in one outbreak. Unlike the clinical signs observed on chicken farms, no signs of the membranes and skin were observed in the duck flocks by farmers nor veterinarians.

### 3.3 Mortality

#### 3.3.1 Chicken farms

**FIGURE 3:**
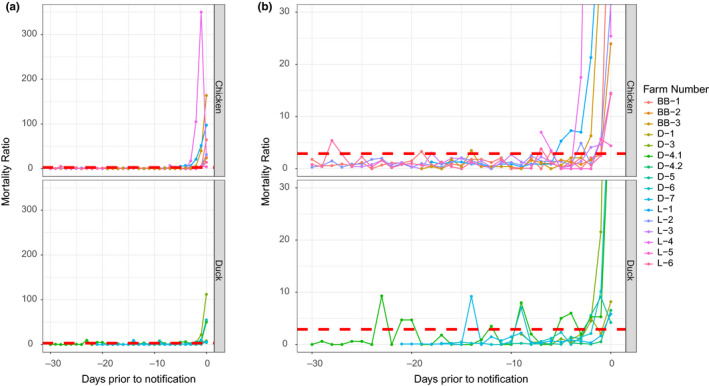
(a) Calculated mortality ratio's in the 30 day period prior to highly pathogenic avian influenza (HPAI) notification for the nine chicken farms (top), of which three broiler breeder (BB) and six layer (L) farms, and six Pekin duck farms (D; bottom). The mortality ratio (MR) threshold of 2.9 is shown in red. In one outbreak on a duck farm, 2 houses were affected: D‐4.1 and D‐4.2. (b) A more detailed plot of the calculated mortality ratio's in the 30 day period prior to HPAI notification in chicken and Pekin duck outbreaks. Cut‐off on the *Y*‐axis was set to 30 to better visualize the behaviour of the MR in days prior to notification. The MR threshold of 2.9 is shown in red

For chicken flock L‐1, the MR exceeded the threshold 5 days prior to the day of notification, whereas in all others outbreaks the MR exceeded the threshold three or fewer days prior to notification (Figure 1). On the day of notification, the MR of all chicken farms exceeded the threshold. The DM exceeded the 0.5% threshold in two farms 1 day prior to notification and in six farms at day of notification. The MR exceeded 2.9 for only a single day on eight occasions on six different farms in the 30 days period prior to notification (Figure 3).

In five out of nine outbreaks, the MR exceeded the proposed threshold prior to observing of clinical signs by the farmer, in three out of nine outbreaks the increase of the MR and first observation of clinical sign coincided, and in one outbreak the clinical signs were observed prior to an increased MR (Figure 1).

#### 3.3.2 Pekin duck farms

The MR exceeded the threshold the first time 5 days prior to day of notification in one house of one Pekin duck farm (D‐4.1; Figure 1). At the day of notification, the MR exceeded the threshold in all six outbreaks for which a MR was available. The DM exceeded 0.5% in four of seven outbreaks on day of notification and only in one outbreak (D‐3) mortality exceeded 0.5% 1 day prior to notification (Figure 1). The MR exceeded 2.9 for only a single day on seven occasions on four different farms in the 30 days period prior to the notification (Figure 3). On six occasions the MR exceeded the threshold for a single day and was <2.9 the following day. On one occasion, the MR exceeded the threshold on two consecutive days in one house of a farm (D‐4.1). This house also had the most occasions (five out of seven) in which the MR temporarily exceeded the threshold.

The MR exceeded the threshold in three out of six outbreaks prior to observation of clinical signs. In one outbreak, the increase of MR coincided with the first observation of clinical signs, and in two out of six outbreaks the clinical signs were observed prior to an increase in MR (Figure 1).

## 4 DISCUSSION

The purpose of this case series was to describe the observed clinical signs in HPAI H5N8 and H5N6 outbreaks on chicken and Pekin duck farms between 2014 and 2018 in the Netherlands and compare this with the onset of an increased MR threshold (Gonzales & Elbers, 2018). We describe that in 12 out of 15 outbreaks for which a MR was available on chicken and Pekin duck farms, the MR increase preceded or coincided with the first observation of clinical signs by the farmer. In one chicken and two Pekin duck outbreaks, clinical signs were observed prior to a MR increase. Additionally, in most cases the first clinical signs were seen within a day or two after the onset of an increased MR. Although these observations conveyed the idea that MR could be an earlier indicator of HPAI infection, when MR is less affected, for instance for less virulent AI virus strains, the observation of clinical signs in combination with MR may provide additional indication for farmers and veterinarians and prompt them to notifying the disease.

It should be noted that we looked at the first day the MR exceeded the threshold and compared that with the first observation of clinical signs according to the interviews with the farmers, because we were interested in the timing of detection of clinical signs in relation to an increase of the MR. Gonzales and Elbers (2018), however, proposed that the MR should be implemented in practice to notify authorities only after the MR exceeds the threshold for two consecutive days, to reduce false‐positive signals (i.e. increase specificity). By using that logic, an increased MR still preceded or coincided with the first observation of clinical signs in eight out of 15 outbreaks (five outbreaks on chicken farms, three outbreaks on Pekin duck farms).

To our knowledge, this is the first report to apply this MR threshold in Pekin duck outbreaks. Our results show that the MR fluctuated more in Pekin duck farms in comparison with the layer farms and exceeded the threshold more often in the 30 day period prior to the HPAI virus infection. However, in six of the seven occasions that the MR exceeded the threshold in Pekin duck farms, the MR only exceeded the set threshold for 1 day, which would not lead to a notification to the authorities when the MR is applied as suggested by Gonzales and Elbers (2018). Furthermore, the MR exceeded the threshold in all outbreaks on Pekin duck and chicken farms, whereas the DM only exceeded 0.5% in four out of seven outbreaks on the Pekin duck farms and in six out of nine outbreaks on chicken farms. Moreover, in eight out of nine outbreaks in chicken and Pekin duck farms where the DM did exceed 0.5%, the MR had already exceeded its set thresholds 1–4 days prior. In pheasants, however, it was found that exceeding a set absolute threshold on two consecutive days resulted in the best trade‐off between false‐alarm rate and early detection compared with a 7 day moving average or exceeding a set absolute threshold for 1 day (Ssematimba et al., 2019). Due to the limited data set, we were not able to evaluate these trade‐offs appropriately, but the results obtained from these H5Nx outbreaks in the Netherlands suggest that the MR could be a more sensitive parameter to monitor for HPAI virus infection in Pekin ducks compared with the current DM used in Dutch legislation for notification to the authorities. As the choice of an effective mortality threshold requires evaluation of the trade‐off between lowering the threshold to enhance early detection of infected flocks and the corresponding increase in false alarm rates in uninfected flocks, more research is needed. To assess and validate the currently used MR, and determine the best set threshold for an optimal sensitivity and specificity for Pekin ducks, and where possible also for other poultry species, flock data from outbreaks with preferably different HPAI virus strains should be analysed.

In chickens, veterinarians reported general clinical signs in all nine outbreaks, signs of the gastrointestinal tract in seven outbreaks, mucosal membrane and skin in five and respiratory tract also in five outbreaks at the day of notification. The clinical sings were not notably different over the years, although the outbreaks in 2014 and 2016 were caused by subtype H5N8 and in 2017–2018 by H5N6. These findings are in line with earlier reports about H5Nx infections in chickens (Pohlmann et al., 2017; Sun et al., 2016). Sun et al. (2016) found that naturally infected H5Nx chickens developed systemic disease, congestion and haemorrhage of the comb, wattles and feet, subcutaneous haemorrhages and oedema around the hock and shanks, which are similar to the clinical signs that were reported in the mucosal membrane and skin (Table S1). Early in the flock infection process, however, the farmers in our study mainly observed clinical signs that could only be considered as general clinical signs, which are not specific for HPAI virus infection (Elbers et al., 2007; Swayne, Suarez, & Sims, 2013) suggesting that in early stages of the infection process it is difficult to distinguish HPAI virus infection from other diseases that lead to systemic disease. Clearly, when the farmer or veterinarian suspects HPAI infection, immediate notification is needed. However, in cases with rather mild clinical signs or limited increased mortality not specific for HPAI, the submission of cloacal or pharyngeal swabs to exclude AI infection is recommended to facilitate detection of circulating AI virus at an early stage. This is already implemented in the Netherlands, as mentioned in Box 1, and has shown to be effective in detecting LPAI outbreaks, and incidentally, as described in this study for duck farm D‐1, also for detection of HPAI outbreaks at an early stage (Elbers, Gorgievski‐Duijvesteijn, Zarafshani, & Koch, 2010).

In Pekin ducks, veterinarians reported general clinical signs and nervous or locomotor signs most often and in all outbreaks. This was followed by respiratory and gastrointestinal signs, which were both reported in five out of seven outbreaks. The high incidence of nervous and locomotor signs, also observed by six of the Pekin duck farmers, is in contrast with the incidence in chickens, where nervous and locomotor signs were only reported in two outbreaks by veterinarians. Although the outbreaks in 2014 and 2016 were caused by different subtypes of H5Nx, the clinical signs were not notably different over the years in Pekin ducks. The observation of neurological signs (mainly head tremors, torticollis and ataxia) in our study in Pekin ducks is in line with findings reported in an outbreak of H5N8 among fattening Pekin ducks in Hungary in 2015 (Bányai et al., 2016) where affected ducks showed neurologic signs, including torticollis. These findings are further supported with the results of studies where Pekin ducks were infected experimentally with different H5Nx subtypes of clade 2.3.4.4 (Sun et al., 2016). However, in other experimental inoculated domestic ducks with H5N8 viruses of the same clade (2.3.4.4), a wide range of pathobiological outcomes, from no clinical signs to some neurological signs to severe disease, were reported (Kang et al., 2015; Pantin‐Jackwood et al., 2017; Shivaprasad, Carnaccini, Crossley, Senties‐Cue, & Chin, 2016). Although previous cases have shown that clinical manifestation and mortality in Anseriformes species highly depends on the phenotypic characteristics of the HPAI virus (EFSA Panel on Animal Health and Welfare (EFSA AHAW Panel), 2017), the current case series emphasizes that Pekin duck farmers and veterinarians should be aware that observation of neurological signs in a flock could be an indication of HPAI virus infection and might require further diagnostic follow‐up.

Compared to the veterinarians, farmers observed and reported less specific clinical signs, especially regarding respiratory and gastrointestinal signs in both chicken and ducks. This difference may be due to the specialized training and experience of the veterinarians in poultry veterinary medicine to observe signs of disease, and veterinarians may be better equipped with a repertoire of specific words to indicate their observations and relate that to a specific organ system. The discrepancy in observation of clinical signs between farmers and veterinarians is, however, smaller than we anticipated, suggesting that the farmers were aware of signs to look for. This shows that training and awareness of the farmer in detecting clinical signs is an important tool in detecting HPAI virus infection at an early stage.

The willingness of the farmer and practitioners to report a suspicion of a notifiable disease to the authorities may be different for the very first suspicion compared with suspicions after the first confirmed HPAI outbreak (Elbers et al., 2010). To prevent the spread of HPAI viruses to other farms, it is crucial to notify a suspicion as early as possible to be able to adequately diagnose and quickly depopulate the farms. The first outbreak of a HPAI (H5Nx; outbreak no. L‐1) in 2014 had increased mortality (>2.9) for 5 days prior to notification. In the outbreaks after 2014, the mortality ratios exceeded the threshold 0–3 days prior to notification, which suggest that farmers were more alert and reported a suspicion of notifiable disease more rapidly. Additionally, two Pekin duck farms and one chicken farm had multiple outbreaks of HPAI in 2014, 2016–2017 on their farms, making the farmers even more aware of the risk of a new outbreak. Due to the fast reporting of HPAI suspicion of farmers and veterinarians to the authorities, the spread of HPAI viruses to other poultry facilities was minimized.

To conclude, the current study gives an indication that the use of an objective MR with a set threshold could be a reliable parameter to detect HPAI virus infection on chicken and Pekin duck farms at an early stage and may perform even better when complemented with detection of clinical signs in poultry farms, provided farmers are well trained to notice them. These results underline the need to validate the MR in Pekin ducks and other poultry species, and it should encourage farmers, veterinarians and veterinary institutes in other countries to monitor and register mortality on farms more rigorously, because a poultry‐specific MR could serve as an important indicator in HPAI poultry surveillance programs.

## CONFLICT OF INTEREST

The authors declare no conflict of interest.

## Supporting information

Table S1–S4

## Data Availability

Most of the data that support the findings of this study are available in the article or supplementary materials. Additional data are available on request from the corresponding author. The data are not publicly available due to privacy restrictions.
