# Supplementary material for: Highly pathogenic avian influenza subtype H5Nx clade 2.3.4.4 outbreaks in Dutch poultry farms, 2014–2018: Clinical signs and mortality
Source: Transbound Emerg Dis. 2020 May 17;68(1):88–97. doi: 10.1111/tbed.13597 (PMC8048556; doi:10.1111/tbed.13597)
Supplement: Supplementary file 1 — Table S1–S4 [file TBED-68-88-s001.docx]

**Supporting Information**

Highly Pathogenic Avian Influenza subtype H5Nx clade 2.3.4.4 outbreaks in Dutch poultry farms, 2014-2018: clinical signs and mortality

Janneke Schreuder^1*^, Thijs T.M. Manders^1*^, Armin R.W. Elbers^2^, Arco N. van der Spek^3^ Ruth J. Bouwstra^4^, J. Arjan Stegeman^1^, Francisca C. Velkers^1^

TABLE S1. Clinical signs observed by veterinary professionals, categorized by organ system, on the day of veterinary inspection on HPAIV infected laying hen (L) and broiler breeder (BB) farms*.*

|  | **Outbreak no.** | | | | | | | | | | | | | | | | |
| --- | --- | --- | --- | --- | --- | --- | --- | --- | --- | --- | --- | --- | --- | --- | --- | --- | --- |
|  | L-1 |  | L-2 |  | BB-3 |  | L-3 |  | L-4 |  | L-5 |  | BB-2 |  | L-6 |  | BB-3 |
| **General clinical signs** | **x** |  | **x** |  | **x** |  | **x** |  | **x** |  | **x** |  | **x** |  | **x** |  | **x** |
| Decreased activity, listlessness, apathy | x |  | x |  | x |  | x |  | x |  | x |  | x |  | x |  | x |
| Depression, lethargy |  |  | x |  | x |  |  |  | x |  | x |  | x |  | x |  | x |
| Reduced vocalization |  |  |  |  |  |  |  |  | x |  | x |  |  |  |  |  |  |
| Warm extremities |  |  | x |  |  |  |  |  | x |  | x |  |  |  | x |  |  |
| Cold extremities |  |  | x |  |  |  |  |  |  |  |  |  |  |  |  |  |  |
| Shivering |  |  |  |  | x |  |  |  |  |  |  |  |  |  |  |  |  |
| Reduced feed intake |  |  |  |  |  |  |  |  |  |  | x |  |  |  |  |  |  |
| Reduced water intake |  |  |  |  |  |  |  |  |  |  |  |  |  |  |  |  |  |
| Increased water intake |  |  |  |  |  |  |  |  |  |  |  |  |  |  |  |  |  |
| Decreased daily growth |  |  |  |  |  |  |  |  |  |  |  |  |  |  |  |  |  |
| Hunched posture |  |  |  |  |  |  |  |  |  |  |  |  |  |  |  |  |  |
| Ruffled feathers | x |  | x |  | x |  | x |  | x |  | x |  | x |  | x |  | x |
| Closed eyes |  |  |  |  |  |  |  |  | x |  |  |  |  |  |  |  | x |
| Sudden death | x |  | x |  | x |  | x |  | x |  |  |  | x |  | x |  | x |
|  |  |  |  |  |  |  |  |  |  |  |  |  |  |  |  |  |  |
| **Clinical signs attributed to** |  |  |  |  |  |  |  |  |  |  |  |  |  |  |  |  |  |
| **Nervous or locomotor system** |  |  |  |  | **x** |  |  |  |  |  |  |  | **x** |  |  |  |  |
| Tremors |  |  |  |  |  |  |  |  |  |  |  |  |  |  |  |  |  |
| Paralysis |  |  |  |  |  |  |  |  |  |  |  |  |  |  |  |  |  |
| Torticollis |  |  |  |  |  |  |  |  |  |  |  |  |  |  |  |  |  |
| Opisthotonus |  |  |  |  | x |  |  |  |  |  |  |  |  |  |  |  |  |
| Abnormal gait |  |  |  |  |  |  |  |  |  |  |  |  |  |  |  |  |  |
| Lameness |  |  |  |  |  |  |  |  |  |  |  |  |  |  |  |  |  |
| Ataxia |  |  |  |  |  |  |  |  |  |  |  |  |  |  |  |  |  |
| Inability to stand |  |  |  |  |  |  |  |  |  |  |  |  |  |  |  |  |  |
|  |  |  |  |  |  |  |  |  |  |  |  |  |  |  |  |  |  |
| **Mucosal membranes and skin** |  |  | **x** |  | **x** |  |  |  | **x** |  |  |  | **x** |  |  |  | **x** |
| Cyanosis of the wattles |  |  |  |  | x |  |  |  | x |  |  |  | x |  |  |  | x |
| Haemorrhagic conjunctiva |  |  | x |  | x |  |  |  |  |  |  |  |  |  |  |  |  |
| Swollen head |  |  |  |  |  |  |  |  |  |  |  |  | x |  |  |  |  |
| Swollen wattles |  |  |  |  |  |  |  |  | x |  |  |  | x |  |  |  | x |
| Dark small combs |  |  | x |  | x |  |  |  |  |  |  |  |  |  |  |  | x |
|  |  |  |  |  |  |  |  |  |  |  |  |  |  |  |  |  |  |
| **Respiratory tract** |  |  | **x** |  | **x** |  | **x** |  |  |  |  |  | **x** |  |  |  | **x** |
| Rales/rattles |  |  |  |  |  |  |  |  |  |  |  |  |  |  |  |  |  |
| Sneezing |  |  |  |  |  |  |  |  |  |  |  |  |  |  |  |  |  |
| Excessive lacrimation |  |  |  |  |  |  |  |  |  |  |  |  |  |  |  |  |  |
| Nasal discharge |  |  |  |  |  |  |  |  |  |  |  |  |  |  |  |  |  |
| Sinusitis |  |  |  |  |  |  |  |  |  |  |  |  |  |  |  |  |  |
| Conjunctivitis |  |  |  |  | x |  | x |  |  |  |  |  | x |  |  |  | x |
|  |  |  |  |  |  |  |  |  |  |  |  |  |  |  |  |  |  |
| **Gastrointestinal tract** |  |  | **x** |  | **x** |  |  |  | **x** |  | **x** |  | **x** |  | **x** |  | **x** |
| Diarrhoea |  |  | x |  | x |  |  |  | x |  | x |  | x |  | x |  | x |
|  |  |  |  |  |  |  |  |  |  |  |  |  |  |  |  |  |  |
| **Reproduction tract** |  |  | **x** |  |  |  |  |  | **x** |  | **x** |  |  |  | **x** |  |  |
| Decreased egg production |  |  |  |  |  |  |  |  | x |  | x |  |  |  | x |  |  |
| Decreased egg-shell quality |  |  |  |  |  |  |  |  |  |  | x |  |  |  |  |  |  |
| Pale or mottled eggs |  |  | x |  |  |  |  |  | x |  | x |  |  |  |  |  |  |

Table S2. Clinical signs observed by veterinary professionals, categorized by organ system, on the day of veterinary inspection on HPAIV infected Pekin duck (D) farms*.*

|  | **Outbreak no.** | | | | | | | | | | | | |
| --- | --- | --- | --- | --- | --- | --- | --- | --- | --- | --- | --- | --- | --- |
|  | D - 1 |  | D - 2 |  | D - 3 |  | D -4 |  | D - 5 |  | D -6 |  | D - 7 |
| **General clinical signs** | **x** |  | **x** |  | **x** |  | **x** |  | **x** |  | **x** |  | **x** |
| Decreased activity, listlessness, apathy | x |  | x |  | x |  | x |  | x |  | x |  | x |
| Depression, lethargy |  |  | x |  | x |  | x |  | x |  |  |  | x |
| Reduced vocalization |  |  | x |  |  |  |  |  |  |  | x |  |  |
| Warm extremities |  |  |  |  |  |  |  |  |  |  | x |  |  |
| Cold extremities |  |  | x |  | x |  |  |  |  |  |  |  |  |
| Shivering |  |  |  |  |  |  |  |  |  |  |  |  |  |
| Reduced feed intake |  |  |  |  |  |  |  |  |  |  | x |  |  |
| Reduced water intake |  |  |  |  |  |  |  |  |  |  |  |  |  |
| Increased water intake |  |  |  |  |  |  |  |  |  |  |  |  |  |
| Decreased daily growth |  |  |  |  |  |  |  |  |  |  |  |  |  |
| Hunched posture |  |  |  |  |  |  |  |  |  |  |  |  |  |
| Ruffled feathers |  |  |  |  |  |  | x |  |  |  | x |  |  |
| Closed eyes |  |  |  |  |  |  |  |  |  |  |  |  |  |
| Sudden death |  |  | x |  |  |  |  |  |  |  | x |  | x |
|  |  |  |  |  |  |  |  |  |  |  |  |  |  |
| **Clinical signs attributed to** |  |  |  |  |  |  |  |  |  |  |  |  |  |
| **Nervous or locomotor system** | **x** |  | **x** |  | **x** |  | **x** |  | **x** |  | **x** |  | **x** |
| Tremors | x |  | x |  | x |  |  |  | x |  |  |  | x |
| Paralysis |  |  |  |  |  |  |  |  |  |  |  |  |  |
| Torticollis |  |  | x |  | x |  | x |  |  |  |  |  |  |
| Opisthotonus |  |  | x |  |  |  |  |  |  |  |  |  |  |
| Abnormal gait |  |  |  |  |  |  |  |  |  |  |  |  |  |
| Lameness |  |  |  |  |  |  |  |  |  |  |  |  | x |
| Ataxia |  |  | x |  |  |  |  |  | x |  |  |  | x |
| Inability to stand |  |  |  |  |  |  |  |  |  |  |  |  | x |
|  |  |  |  |  |  |  |  |  |  |  |  |  |  |
| **Mucosal membranes and skin** |  |  |  |  |  |  |  |  |  |  |  |  |  |
| Cyanosis of the wattles |  |  |  |  |  |  |  |  |  |  |  |  |  |
| Haemorrhagic conjunctiva |  |  |  |  |  |  |  |  |  |  |  |  |  |
| Swollen head |  |  |  |  |  |  |  |  |  |  |  |  |  |
| Swollen wattles |  |  |  |  |  |  |  |  |  |  |  |  |  |
| Dark small combs |  |  |  |  |  |  |  |  |  |  |  |  |  |
|  |  |  |  |  |  |  |  |  |  |  |  |  |  |
| **Respiratory tract** | **x** |  |  |  | **x** |  |  |  | **x** |  | **x** |  | **x** |
| Rales/rattles |  |  |  |  |  |  |  |  |  |  |  |  |  |
| Sneezing |  |  |  |  |  |  |  |  |  |  |  |  |  |
| Excessive lacrimation |  |  |  |  |  |  |  |  |  |  | x |  | x |
| Nasal discharge | x |  |  |  |  |  |  |  |  |  |  |  |  |
| Sinusitis |  |  |  |  |  |  |  |  |  |  |  |  |  |
| Conjunctivitis | x |  |  |  | x |  |  |  |  |  | x |  | x |
|  |  |  |  |  |  |  |  |  | x |  |  |  |  |
| **Gastrointestinal tract** |  |  | **x** |  | **x** |  |  |  | **x** |  | **x** |  | **x** |
| Diarrhoea |  |  | x |  | x |  |  |  | x |  | x |  | x |
|  |  |  |  |  |  |  |  |  |  |  |  |  |  |
| **Reproduction tract** |  |  |  |  |  |  |  |  |  |  |  |  |  |
| Decreased egg production |  |  |  |  |  |  |  |  |  |  |  |  |  |
| Decreased egg-shell quality |  |  |  |  |  |  |  |  |  |  |  |  |  |
| Pale or mottled eggs |  |  |  |  |  |  |  |  |  |  |  |  |  |

Table S3. Clinical signs observed by the farmers, categorized by organ system, for the HPAIV infected laying hen (L) and broiler breeder (BB) farms in the three day period prior to notification.

|  | **Outbreak no.** | | | | | | | | | | | | | | | | | | | | | | | | | | | | | | | | | | | |
| --- | --- | --- | --- | --- | --- | --- | --- | --- | --- | --- | --- | --- | --- | --- | --- | --- | --- | --- | --- | --- | --- | --- | --- | --- | --- | --- | --- | --- | --- | --- | --- | --- | --- | --- | --- | --- |
|  | L- 1 | | | | L -2 | | | | BB - 1 | | | | L - 3 | | | | L- 4 | | | | L - 5 | | | | BB - 2 | | | | L - 6 | | | | BB - 3 | | | |
|  | Days prior to notification | | | | Days prior to notification | | | | Days prior to notification | | | | Days prior to notification | | | | Days prior to notification | | | | Days prior to notification | | | | Days prior to notification | | | | Days prior to notification | | | | Days prior to notification | | | |
|  | 0 | 1 | 2 | 3 | 0 | 1 | 2 | 3 | 0 | 1 | 2 | 3 | 0 | 1 | 2 | 3 | 0 | 1 | 2 | 3 | 0 | 1 | 2 | 3 | 0 | 1 | 2 | 3 | 0 | 1 | 2 | 3 | 0 | 1 | 2 | 3 |
| **General clinical signs** | **x** | **x** |  |  | **x** | **x** |  |  | **x** |  |  |  | **x** | **x** | **x** | **x** | **x** |  |  |  | **x** |  |  |  | **x** |  |  |  | **x** |  |  |  | **x** | **x** |  |  |
| Decreased activity, listlessness, apathy | x | x |  |  | x | x |  |  | x |  |  |  | x | x | x | x | x |  |  |  | x |  |  |  | x |  |  |  | x |  |  |  | x | x |  |  |
| Depression, lethargy |  |  |  |  |  |  |  |  |  |  |  |  |  |  |  |  | x |  |  |  | x |  |  |  |  |  |  |  |  |  |  |  | x |  |  |  |
| Reduced vocalization |  |  |  |  |  |  |  |  |  |  |  |  |  |  |  |  | x |  |  |  |  |  |  |  |  |  |  |  |  |  |  |  |  |  |  |  |
| Warm extremities |  |  |  |  |  |  |  |  |  |  |  |  |  |  |  |  | x |  |  |  |  |  |  |  | x |  |  |  | x |  |  |  |  |  |  |  |
| Cold extremities |  |  |  |  |  |  |  |  |  |  |  |  | x |  |  |  |  |  |  |  |  |  |  |  |  |  |  |  |  |  |  |  |  |  |  |  |
| Shivering |  |  |  |  | x | x |  |  |  |  |  |  |  |  |  |  |  |  |  |  |  |  |  |  |  |  |  |  |  |  |  |  |  |  |  |  |
| Reduced feed intake |  |  |  |  |  |  |  |  |  |  |  |  |  |  |  |  | x |  |  |  | x |  |  |  |  |  |  |  |  |  |  |  |  |  |  |  |
| Reduced water intake |  |  |  |  |  |  |  |  |  |  |  |  |  |  |  |  |  |  |  |  |  |  |  |  |  |  |  |  |  |  |  |  |  |  |  |  |
| Increased water intake |  |  |  |  |  |  |  |  |  |  |  |  |  |  |  |  |  |  |  |  |  |  |  |  | x |  |  |  |  |  |  |  |  |  |  |  |
| Decreased daily growth |  |  |  |  |  |  |  |  |  |  |  |  |  |  |  |  |  |  |  |  |  |  |  |  |  |  |  |  |  |  |  |  |  |  |  |  |
| Hunched posture |  |  |  |  |  |  |  |  |  |  |  |  |  |  |  |  |  |  |  |  |  |  |  |  |  |  |  |  |  |  |  |  |  |  |  |  |
| Ruffled feathers | x | x |  |  |  |  |  |  |  |  |  |  |  |  |  |  | x |  |  |  | x |  |  |  | x |  |  |  | x |  |  |  | x | x |  |  |
| Closed eyes |  |  |  |  |  |  |  |  |  |  |  |  | x |  |  |  | x |  |  |  |  |  |  |  |  |  |  |  | x |  |  |  |  |  |  |  |
| Sudden death | x | x |  |  | x |  |  |  | x |  |  |  |  |  |  |  | x |  |  |  | x |  |  |  | x |  |  |  | x |  |  |  |  |  |  |  |
|  |  |  |  |  |  |  |  |  |  |  |  |  |  |  |  |  |  |  |  |  |  |  |  |  |  |  |  |  |  |  |  |  |  |  |  |  |
| **Clinical signs attributed to** |  |  |  |  |  |  |  |  |  |  |  |  |  |  |  |  |  |  |  |  |  |  |  |  |  |  |  |  |  |  |  |  |  |  |  |  |
| **Nervous or locomotor system** |  |  |  |  |  |  |  |  |  |  |  |  |  |  |  |  |  |  |  |  |  |  |  |  |  |  |  |  |  |  |  |  |  |  |  |  |
| Tremors |  |  |  |  |  |  |  |  |  |  |  |  |  |  |  |  |  |  |  |  |  |  |  |  |  |  |  |  |  |  |  |  |  |  |  |  |
| Paralysis |  |  |  |  |  |  |  |  |  |  |  |  |  |  |  |  |  |  |  |  |  |  |  |  |  |  |  |  |  |  |  |  |  |  |  |  |
| Torticollis |  |  |  |  |  |  |  |  |  |  |  |  |  |  |  |  |  |  |  |  |  |  |  |  |  |  |  |  |  |  |  |  |  |  |  |  |
| Opisthotonus |  |  |  |  |  |  |  |  |  |  |  |  |  |  |  |  |  |  |  |  |  |  |  |  |  |  |  |  |  |  |  |  |  |  |  |  |
| Abnormal gait |  |  |  |  |  |  |  |  |  |  |  |  |  |  |  |  |  |  |  |  |  |  |  |  |  |  |  |  |  |  |  |  |  |  |  |  |
| Lameness |  |  |  |  |  |  |  |  |  |  |  |  |  |  |  |  |  |  |  |  |  |  |  |  |  |  |  |  |  |  |  |  |  |  |  |  |
| Ataxia |  |  |  |  |  |  |  |  |  |  |  |  |  |  |  |  |  |  |  |  |  |  |  |  |  |  |  |  |  |  |  |  |  |  |  |  |
| Inability to stand |  |  |  |  |  |  |  |  |  |  |  |  |  |  |  |  |  |  |  |  |  |  |  |  |  |  |  |  |  |  |  |  |  |  |  |  |
|  |  |  |  |  |  |  |  |  |  |  |  |  |  |  |  |  |  |  |  |  |  |  |  |  |  |  |  |  |  |  |  |  |  |  |  |  |
| **Mucosal membranes and skin** |  |  |  |  |  |  |  |  |  |  |  |  |  |  |  |  | **x** |  |  |  |  |  |  |  | **x** |  |  |  |  |  |  |  | **x** |  |  |  |
| Cyanosis of the wattles |  |  |  |  |  |  |  |  |  |  |  |  |  |  |  |  | x |  |  |  |  |  |  |  | x |  |  |  |  |  |  |  | x |  |  |  |
| Haemorrhagic conjunctiva |  |  |  |  |  |  |  |  |  |  |  |  |  |  |  |  |  |  |  |  |  |  |  |  |  |  |  |  |  |  |  |  |  |  |  |  |
| Swollen head |  |  |  |  |  |  |  |  |  |  |  |  |  |  |  |  | x |  |  |  |  |  |  |  | x |  |  |  |  |  |  |  |  |  |  |  |
| Swollen wattles |  |  |  |  |  |  |  |  |  |  |  |  |  |  |  |  | x |  |  |  |  |  |  |  | x |  |  |  |  |  |  |  |  |  |  |  |
| Dark small combs |  |  |  |  |  |  |  |  |  |  |  |  |  |  |  |  |  |  |  |  |  |  |  |  |  |  |  |  |  |  |  |  |  |  |  |  |
|  |  |  |  |  |  |  |  |  |  |  |  |  |  |  |  |  |  |  |  |  |  |  |  |  |  |  |  |  |  |  |  |  |  |  |  |  |
| **Respiratory tract** |  |  |  |  |  |  |  |  |  |  |  |  | **x** |  |  |  |  |  |  |  |  |  |  |  |  |  |  |  |  |  |  |  | **x** |  |  |  |
| Rales/rattles |  |  |  |  |  |  |  |  |  |  |  |  |  |  |  |  |  |  |  |  |  |  |  |  |  |  |  |  |  |  |  |  |  |  |  |  |
| Sneezing |  |  |  |  |  |  |  |  |  |  |  |  |  |  |  |  |  |  |  |  |  |  |  |  |  |  |  |  |  |  |  |  |  |  |  |  |
| Excessive lacrimation |  |  |  |  |  |  |  |  |  |  |  |  | x |  |  |  |  |  |  |  |  |  |  |  |  |  |  |  |  |  |  |  |  |  |  |  |
| Nasal discharge |  |  |  |  |  |  |  |  |  |  |  |  |  |  |  |  |  |  |  |  |  |  |  |  |  |  |  |  |  |  |  |  | x |  |  |  |
| Sinusitis |  |  |  |  |  |  |  |  |  |  |  |  |  |  |  |  |  |  |  |  |  |  |  |  |  |  |  |  |  |  |  |  |  |  |  |  |
| Conjunctivitis |  |  |  |  |  |  |  |  |  |  |  |  |  |  |  |  |  |  |  |  |  |  |  |  |  |  |  |  |  |  |  |  |  |  |  |  |
|  |  |  |  |  |  |  |  |  |  |  |  |  |  |  |  |  |  |  |  |  |  |  |  |  |  |  |  |  |  |  |  |  |  |  |  |  |
| **Gastrointestinal tract** |  |  |  |  |  |  |  |  |  |  |  |  |  |  |  |  | **x** |  |  |  | **x** |  |  |  |  |  |  |  | **x** |  |  |  |  |  |  |  |
| Diarrhoea |  |  |  |  |  |  |  |  |  |  |  |  |  |  |  |  | x |  |  |  | x |  |  |  |  |  |  |  | x |  |  |  |  |  |  |  |
|  |  |  |  |  |  |  |  |  |  |  |  |  |  |  |  |  |  |  |  |  |  |  |  |  |  |  |  |  |  |  |  |  |  |  |  |  |
| **Reproduction tract** |  |  |  |  | **x** |  |  |  | **x** |  |  |  | **x** |  |  |  | **x** |  |  |  | **x** |  |  |  |  |  |  |  | **x** |  |  |  |  |  |  |  |
| Decreased egg production |  |  |  |  |  |  |  |  |  |  |  |  |  |  |  |  | x |  |  |  | x |  |  |  |  |  |  |  | x |  |  |  |  |  |  |  |
| Decreased egg-shell quality |  |  |  |  |  |  |  |  |  |  |  |  | x |  |  |  | x |  |  |  | x |  |  |  |  |  |  |  |  |  |  |  |  |  |  |  |
| Pale or mottled eggs |  |  |  |  | x |  |  |  | x |  |  |  |  |  |  |  | x |  |  |  | x |  |  |  |  |  |  |  |  |  |  |  |  |  |  |  |

Table S4. Clinical signs observed by the farmers, categorized by organ system, for the HPAIV infected Pekin duck (D) farms in the three day period prior to notification.

|  | **Outbreak no.** | | | | | | | | | | | | | | | | | | | | |
| --- | --- | --- | --- | --- | --- | --- | --- | --- | --- | --- | --- | --- | --- | --- | --- | --- | --- | --- | --- | --- | --- |
|  | D - 1 | | | D - 2 | | | D -3 | | | D -4 | | | D -5 | | | D -6 | | | D -7 | | |
|  | Days prior to notification | | | Days prior to notification | | | Days prior to notification | | | Days prior to notification | | | Days prior to notification | | | Days prior to notification | | | Days prior to notification | | |
|  | 0 | 1 | 2 | 0 | 1 | 2 | 0 | 1 | 2 | 0 | 1 | 2 | 0 | 1 | 2 | 0 | 1 | 2 | 0 | 1 | 2 |
| **General clinical signs** |  |  | **x** | **x** |  |  | **x** | **x** |  | **x** |  |  | **x** |  |  | **x** |  |  | **x** | **x** |  |
| Decreased activity, listlessness, apathy |  |  | x | x |  |  | x | x |  | x |  |  | x |  |  | x |  |  | x | x |  |
| Depression, lethargy |  |  |  | x |  |  | x |  |  | x |  |  | x |  |  |  |  |  |  |  |  |
| Reduced vocalization |  |  |  | x |  |  |  |  |  |  |  |  |  |  |  |  |  |  |  |  |  |
| Warm extremities |  |  |  |  |  |  |  |  |  |  |  |  |  |  |  |  |  |  |  |  |  |
| Cold extremities |  |  |  |  |  |  |  |  |  |  |  |  |  |  |  |  |  |  |  |  |  |
| Shivering |  |  |  |  |  |  |  |  |  |  |  |  |  |  |  |  |  |  |  |  |  |
| Reduced feed intake |  |  |  |  |  |  |  |  |  |  |  |  |  |  |  | x |  |  |  |  |  |
| Reduced water intake |  |  |  |  |  |  |  |  |  |  |  |  |  |  |  |  |  |  |  |  |  |
| Increased water intake |  |  |  |  |  |  |  |  |  |  |  |  |  |  |  |  |  |  |  |  |  |
| Decreased daily growth |  |  | x |  |  |  |  |  |  |  |  |  |  |  |  |  |  |  |  |  |  |
| Hunched posture |  |  |  |  |  |  |  |  |  |  |  |  |  |  |  |  |  |  |  |  |  |
| Ruffled feathers |  |  |  |  |  |  |  |  |  |  |  |  |  |  |  |  |  |  |  |  |  |
| Closed eyes |  |  |  |  |  |  |  |  |  |  |  |  |  |  |  |  |  |  |  |  |  |
| Sudden death |  |  |  | x |  |  |  |  |  | x |  |  |  |  |  | x |  |  |  |  |  |
|  |  |  |  |  |  |  |  |  |  |  |  |  |  |  |  |  |  |  |  |  |  |
| **Clinical signs attributed to** |  |  |  |  |  |  |  |  |  |  |  |  |  |  |  |  |  |  |  |  |  |
| **Nervous or locomotor system** |  |  | **x** | **x** |  |  | **x** |  |  | **x** |  |  | **x** | **x** | **x** |  |  |  | **x** | **x** |  |
| Tremors |  |  | x |  |  |  | x |  |  |  |  |  | x | x | x |  |  |  | x |  |  |
| Paralysis |  |  |  |  |  |  |  |  |  |  |  |  |  |  |  |  |  |  |  |  |  |
| Torticollis |  |  |  |  |  |  | x |  |  | x |  |  |  |  |  |  |  |  |  |  |  |
| Opisthotonus |  |  |  | x |  |  |  |  |  |  |  |  |  |  |  |  |  |  |  |  |  |
| Abnormal gait |  |  |  |  |  |  |  |  |  |  |  |  |  |  |  |  |  |  |  |  |  |
| Lameness |  |  |  |  |  |  |  |  |  |  |  |  |  |  |  |  |  |  |  |  |  |
| Ataxia |  |  |  |  |  |  |  |  |  |  |  |  | x |  |  |  |  |  |  |  |  |
| Inability to stand |  |  |  |  |  |  |  |  |  |  |  |  |  |  |  |  |  |  | x | x |  |
|  |  |  |  |  |  |  |  |  |  |  |  |  |  |  |  |  |  |  |  |  |  |
| **Mucosal membranes and skin** |  |  |  |  |  |  |  |  |  |  |  |  |  |  |  |  |  |  |  |  |  |
| Cyanosis of the wattles |  |  |  |  |  |  |  |  |  |  |  |  |  |  |  |  |  |  |  |  |  |
| Haemorrhagic conjunctiva |  |  |  |  |  |  |  |  |  |  |  |  |  |  |  |  |  |  |  |  |  |
| Swollen head |  |  |  |  |  |  |  |  |  |  |  |  |  |  |  |  |  |  |  |  |  |
| Swollen wattles |  |  |  |  |  |  |  |  |  |  |  |  |  |  |  |  |  |  |  |  |  |
| Dark small combs |  |  |  |  |  |  |  |  |  |  |  |  |  |  |  |  |  |  |  |  |  |
|  |  |  |  |  |  |  |  |  |  |  |  |  |  |  |  |  |  |  |  |  |  |
| **Respiratory tract** |  |  | **x** |  |  |  |  |  |  |  |  |  |  |  | **x** |  |  |  |  |  |  |
| Rales/rattles |  |  |  |  |  |  |  |  |  |  |  |  |  |  |  |  |  |  |  |  |  |
| Sneezing |  |  |  |  |  |  |  |  |  |  |  |  |  |  | x |  |  |  |  |  |  |
| Excessive lacrimation |  |  |  |  |  |  |  |  |  |  |  |  |  |  |  |  |  |  |  |  |  |
| Nasal discharge |  |  | x |  |  |  |  |  |  |  |  |  |  |  |  |  |  |  |  |  |  |
| Sinusitis |  |  |  |  |  |  |  |  |  |  |  |  |  |  |  |  |  |  |  |  |  |
| Conjunctivitis |  |  | x |  |  |  |  |  |  |  |  |  |  |  |  |  |  |  |  |  |  |
|  |  |  |  |  |  |  |  |  |  |  |  |  |  |  |  |  |  |  |  |  |  |
| **Gastrointestinal tract** |  |  |  |  |  |  | **x** |  |  |  |  |  | **x** |  |  |  |  |  |  |  |  |
| Diarrhoea |  |  |  |  |  |  | x |  |  |  |  |  | x |  |  |  |  |  |  |  |  |
|  |  |  |  |  |  |  |  |  |  |  |  |  |  |  |  |  |  |  |  |  |  |
| **Reproduction tract** |  |  |  |  |  |  |  |  |  |  |  |  |  |  |  |  |  |  |  |  |  |
| Decreased egg production |  |  |  |  |  |  |  |  |  |  |  |  |  |  |  |  |  |  |  |  |  |
| Decreased egg-shell quality |  |  |  |  |  |  |  |  |  |  |  |  |  |  |  |  |  |  |  |  |  |
| Pale or mottled eggs |  |  |  |  |  |  |  |  |  |  |  |  |  |  |  |  |  |  |  |  |  |
